# Hypertension and hyperparathyroidism are associated with left ventricular hypertrophy in patients on hemodialysis

**DOI:** 10.4103/0971-4065.59337

**Published:** 2009-10

**Authors:** N. Al-Hilali, N. Hussain, A. I. Ataia, M. Al-Azmi, B. Al-Helal, K. V. Johny

**Affiliations:** Department of Medicine, Mubarak Al-Kabeer Hospital, Kuwait

**Keywords:** Hemodialysis, hypertension, hyperparathyroidism, left ventricular hypertrophy

## Abstract

Conflicting data for association between left ventricular hypertrophy (LVH) and secondary hyperparathyroidism has been reported previously among dialysis patients. The present study was conducted to evaluate the association of hyperparathyroidism and hypertension with LVH. Charts of 130 patients on hemodialysis for at least six months were reviewed. All were subjected to M-mode echocardiography. Left ventricular mass (LVM) was calculated by Devereux's formula. LVM Index (LVMI) was calculated by dividing LVM by body surface area. Sera were analyzed for intact parathyroid hormone (iPTH). iPTH of > 32 pmol/l and a mean blood pressure (MAP) of > 107 mmHg were considered high. Patients were stratified into groups according to their MAP and iPTH. A total of (47.7%) patients were males and 68 (52.3%) were females. Their median age was 57 years. The median duration on dialysis was 26 months. Forty eight (36.9%) patients had high BP and 54 (41.5%) had high iPTH. Both high BP and high iPTH were present in 38 (29.2%) patients. Analysis of the relationship between LVM, LVMI, MAP and iPTH showed that LVM and LVMI were significantly (*P* < 0.001) higher in patients with concomitant high BP and high iPTH. LVMI was significantly higher in patients with high iPTH alone. Concomitant high iPTH and high MAP increase the risk of LVH in hemodialysis patients. High iPTH alone might contribute in escalating LVH. Adequate control of hypertension and hyperparathyroidism might reduce the risk of developing LVH.

## Introduction

Left ventricular hypertrophy (LVH) is frequent in maintenance dialysis patients.[1] Several independent factors contribute to the development of LVH in dialysis patients including fluid overload, anemia, hypertension, hyperparathyroidism and arteriovenous fistula.[1–3] Recently, parathyroid hormone (PTH) has been identified as an important cardio-toxin in end stage renal disease (ESRD). Previous studies have supported the view that high serum PTH serum levels in uremic patients may cause deleterious effects in myocardial metabolism and function.[4] The association between PTH levels and LVH has also been reported by some investigators, with inconsistent results.[2,5–7] Treatment of the contributing factors may result in regression of LVH with the subsequent improvement in patient outcome.[8] Hypertension is frequently (80-90%) seen in patients with end stage renal disease.[9–12] Hypertensive patients in hemodialysis have higher left ventricular mass indices than normotensive patients, but similar to those of no uremic hypertensive patients signifying that hypertension is a crucial factor for LVH development.[13–15]

The present study was conducted to examine the association of hypertension and elevated intact parathyroid hormone in LVH and to evaluate their synergetic effect as risk factors for LVH in hemodialysis patients without other major risk factors.

## Materials and Methods

One hundred and thirty adult patients on maintenance hemodialysis for at least six months were prospectively studied. Patients with severe anemia, coronary artery disease, cardiomyopathies, valvular heart disease, previous parathyroidectomy, and connective tissue diseases were excluded from the study. Informed consent was obtained from each participant.

All patients received their dialysis treatment 3 times per week, 4 h per session (12 h weekly). Polysulfone dialyzers and a bicarbonate bath with a dialysate Ca concentration of 1.25 mmol/L and Na concentration of 136 mmol/L were used for dialysis. The blood flow rate was ≥300 mL/min and the dialysate flow rate was 800 mL/min. All patients were receiving recombinant erythropoietin intravenously 2-3 times postdialysis. No dialyzers are reused in our unit. The dialysis dose as Kt/V was calculated according to DOQI guidelines.[16]

All patients were subjected to M-mode echocardiography. Echocardiograms were performed when the patient reached their estimated ideal weight. Left ventricular diameters and wall thicknesses were measured from 2-dimensional targeted M-mode echocardiography. Left ventricular mass (LVM) was calculated by using Devereux's formula, and was indexed (LVM index) for body surface area,[17,18] considering the diastolic measurements of left ventricular internal diameter (LVID), interventricular septal thickness (IVST) and posterior wall thickness (PWT): LVMI (g/m^2^) 5 (1.04 [(IVST 1 LVID 1 PWT) 3-LVID3]-14 g)/Body surface area. Bodysurface area (BSA) was calculated using the formula: BSA (m^2^) 5 0.007184 3 weight (kg)0.425 3 height(cm)0.725.[19] LVH was defined as LV mass index (g/m^2^) greater than 131 g/m^2^ in men and greater than 100 g/m^2^ in women.[20] Blood pressure was measured by using a standard automatic blood pressure machine of the same for all patients. An average of three measures was taken to calculate the mean arterial blood pressure. The mean arterial blood pressure (MAP) was calculated by the following equation: MAP 5 [(2 3 diastolic) 1 systolic]/3. Mean arterial blood pressure (MAP) of.107 mmHg was considered to be high.

Blood samples were drawn from the arterial side of the vascular access before starting dialysis and prior to heparin administration, in the midweek session after a 48-h dialysis free interval.

Serum levels of intact parathyroid hormone, cholesterol, triglycerides, high density lipoprotein (HDL) and hemoglobin were measured. Sera of the patients were analyzed for intact parathyroid hormone (iPTH). Intact PTH was measured with immune radiometric assay (IRMA) method in the same laboratory. Other biochemical parameters were carried out by standardized clinical laboratory methods.

Patients were stratified into groups according to their MAP and iPTH. Group 1 5 mean BP less than 107 mmHg with iPTH less than 32 pmol/l. Group 2 5 mean BP less than 107 mmHg with iPTH more than 32 pmol/l. Group 3 5 mean BP more than 107 mmHg with iPTH, 32 pmol/l. Group 4 5 mean BP more than 107 mmHg with iPTH more than 32 pmol/l. iPTH of. 32 pmol/l and a mean blood pressure (MAP) of. 107 mmHg were considered to be high for the study.

### Statistical analysis

All analysis was performed using SPSS 14.0 software, (SPSS, Chicago, IL, USA). Results are presented as median and 25^th^, 75^th^ percentiles.

Comparison of medians among multiple groups was analyzed by Kruskal-Wallis Test and Mann Whitney Test as appropriate. Frequencies were compared by cross tabs using Chi-square method. The logistic regression was used to estimate the risk of factors after controlling confounding between them. *P* value < 0.05 was considered significant.

## Results

Clinical parameters of the hemodialysis patients in the present study are summarized in Table 1. Their median age was 57 (41,68). Sixty two patients (47.7%) were men and 68 (52.3%) were women. Diabetic nephropathy was the most common renal disease observed in the study population (35.4%). Biochemical characteristics of both groups are shown in Table 2. All parameters except iPTH were not significant between groups.

**Table 1:** Clinical characteristic of the patients categorized into groups according to mean arterial blood pressure and parathyroid hormone level

Parameters	Group 1 (56)	Group 2 (26)	Group 3 (10)	Group 4 (38)	All (130)
Age (years)	59 (44,70)	59 (43,69)	36 (31,50)	54 (41,65)	57 (41,68)
Gender (%)					
Male	32 (57.1)	10 (38.5)	2 (20)	18 (47.4)	62 (47.7)
Female	24 (42.9)	16 (61.5)	8 (80)	20 (52.6)	68 (52.3)
BMI					
<18.5	4 (7.14)	2 (7.7)	0	6 (15.79)	12 (9.23)
18.6-24.9	32 (57.1)	10 (38.5)	4 (40)	8 (21.05)	54 (41.54)
25-29.9	10 (17.9)	8 (30.77)	4 (40)	10 (26.32)	32 (24.62)
>30	10 (17.9)	6 (23.1)	2 (20)	14 (36.84)	32 (24.62)
Renal disease (%)					
DN	12 (21.4)	14 (53.9)	4 (40)	16 (42.1)	46 (35.4)
CTID	14 (25)	2 (7.7)	4 (40)	14 (36.8)	34 (26.1)
CGN	18 (32.1)	8 (30.78)	0	4 (10.5)	30 (23.1)*
Others	12 (21.4)	2 (7.7)	2 (20)	4 (10.5)	20 (15.4)
Duration on dialysis (months)	28 (12,43)	18 (7,36)	30 (20,84)	26 (6,57)	26 (12,42)
Medications (%)					
BB	45 (80.4)	22 (84.6)	7 (70)	29 (76.3)	103 (79.2)
CCB	40 (71.4)	17 (65.4)	5 (50)	15 (39.5)	77 (59.2)
ACEI/ARB	23 (41.1)	11 (28.9)	4 (40)	10 (26.3)	48 (36.9)
Kt/V	1.3 ± 0.2	1.2 ± 0.2	1.4 ± 0.3	1.2 ± 0.3	1.3 ± 0.2

Results are presented as median and 25^th^, 75^th^ percentiles.

* *P* = 0.007, BMI - Body mass index; CGN - Chronic glomerulonephritis;

DN - Diabetic nephropathy; CTID - Chronic tubuloinerstitial disease; BB - Betablockers; CCB - Calcium channel blockers; ACEI/ARB - Angiotensin converting enzyme inhibitors, or receptor blockers

**Table 2:** Laboratory characteristics

Parameters	Group 1 (56)	Group 2 (26)	Group 3 (10)	Group 4 (38)	All (130)
Hemoglobin	121 ± 12	116 ± 15	1190 ± 12	115 ± 15	127 ± 15
Corrected calcium (Ca) mmol/l	2.38 ± 0.2	2.38 ± 0.1	2.39 ± 0.1	2.50 ± 0.1	2.41 ± 0.15
Phosphorous (P) mmol/l	1.51 ± 0.5	1.45 ± 0.6	1.61 ± 0.4	1.55 ± 0.5	1.52 ± 0.39
CaxP (mmol^2^/l^2^)	3.6 ± 1.0	3.7 ± 0.3	3.5 ± 0.4	3.9 ± 0.5	3.6 ± 0.5
iPTH pmol/l, (25^th^, 75^th^ percentile)	21.9 (19.0, 26.1)	39 (37.3, 61.7)	22 (20.3, 25.5)	56 (47.0, 89.0)*	41.9 (41.3, 77.4)
Cholesterol (mmol/l)	5.1 ± 1.1	5.3 ± 1.3	5.1 ± 1.1	5.3 ± 1.3	5.2 ± 1.2
Triglycerides (mmol/l)	2.2 ± 1.1	1.9 ± 1.3	2.1 ± 1.1	2.3 ± 1.2	2.1 ± 1.2
HDL (mmol/l)	1.1 ± 0.2	1 ± 0.1	1 ± 0.1	1 ± 0.1	1 ± 0.1
Albumin g/l	40 ± 30	40 ± 28	38 ± 32	41 ± 30	40 ± 30

Biochemical data are presented ± SD, PTH results are presented as median and 25^th^, 75^th^ percentiles

* *P* = 0.001

**Figure 1:**
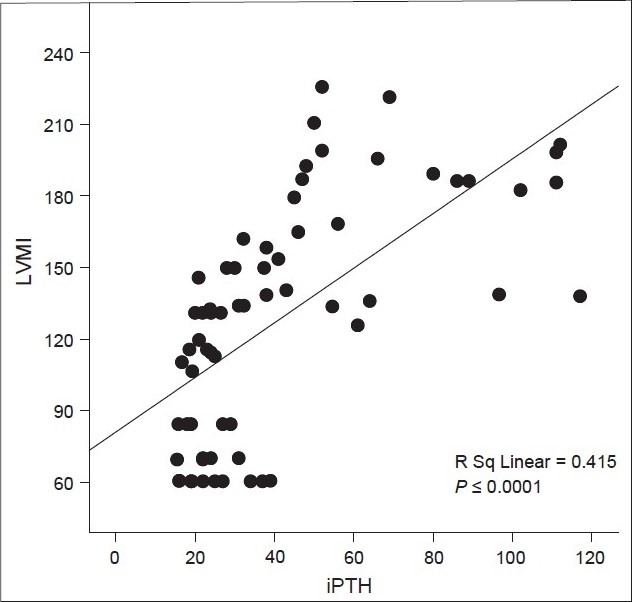
Correlation between intact parathyroid hormone (pmol/l) and left ventricular mass index (g/m^2^) of all patients; iPTH - Intact parathyroid hormone (pmol/l); LVMI - Left ventricular mass index (g/m^2^)

**Figure 2:**
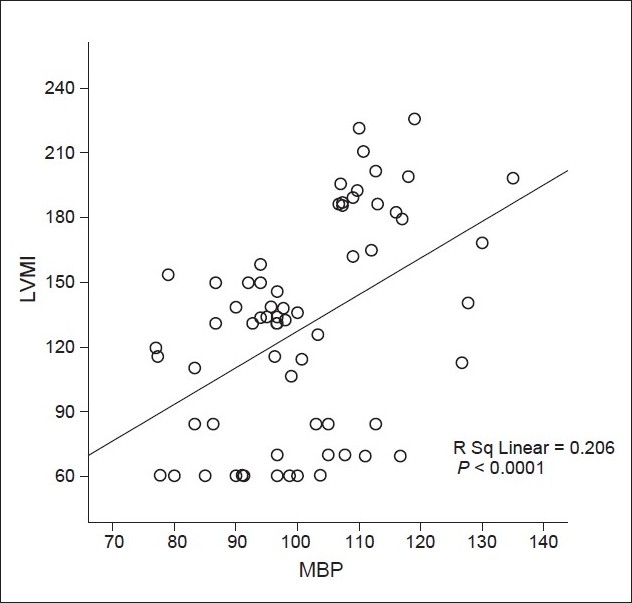
Correlation between mean blood pressure (mmHg) and left ventricular mass index (g/m^2^) of all patients; MAP - Mean blood pressure (mmHg); LVMI - Left ventricular mass index (g/m^2^)

Correlation between iPTH levels and LVM index of all patients are shown in Figure 1. LVM index values correlated positively with iPTH (R Sq 5 0.415, P, 0.0001). Correlation between MAP and LVM index of all patients are shown in Figure 2. LVM index values correlated positively with iPTH (R Sq 5 0.0.206, P, 0.0001).

Table 3 shows the echocardiographic parameters in all groups. There were no significant differences in LVM between Group 1 and Group 2. However, differences between Group 3 and Group 4 and between Group 2 and Group 4 were highly significant. LVMI was significantly higher in Group 2 in comparison to Group 1, Group 4 in comparison to Group 3 and Group 4 in comparison to Group 2. There were also significant differences in IVSD (interventricular septal thickness), between Group 3versus 4 and Group 2 versus Group 4. LVID (left ventricular internal diameter at end diastole) was significantly higher in Group 4 when compared to Group 3 and in Group 4 versus Group 2 and LVPWD (left ventricular wall thickness) was significantly higher in-Group 4 when compared to 2 and Group 4 versus Group 2. As shown in the same table, LVM and LVMI were significantly higher in patients with associated high MAP and high iPTH. Furthermore, LVMI was significantly higher in patients with high iPTH alone. There were no significant differences in LVEF between all groups.

**Table 3:** Echocardiographic parameters

Parameter	Group				*P* value		
		
	1 (56 patients)	2 (26 patients)	3 (10 patients)	4 (38 patients)	Gr 1 *vs.* 2	Gr 3 *vs.* 4	Gr 2 *vs.* 4
LVM, g	175.8 (100.3, 234.4)	220.7 (174.3, 237.5)	114.03 (113.7, 150.4)	327.5 (277.5, 340.4)	0.167	000	000
LVMI, g/m^2^	108.4 (62.9, 131.0)	135.9 (109.4, 141.4)	70.06 (69.5, 91.5)	186.9 (179.3, 198.9)	0.001	000	000
LVEF, %	61.5 (50.5, 67.0)	55.0 (50.0, 68.9)	56.7 (51.0, 69.4)	55.0 (50.0, 67.7)	0.674	0.626	0.641

Results are presented as median and 25^th^, 75^th^ percentiles. LVM (g) - Left ventricular mass; LVMI (g/m^2^), LVEF - Left ventricular ejection fraction

## Discussion

Over the last few years, the severity of coronary artery calcifications (CAC) in ESRD patients has been identified as an independent risk factor for cardiovascular disease. CAC is associated with wide pulse pressure and pulse wave velocity (markers of vascular stiffness) and increased left ventricular mass index.[21–23] Parathormone (PTH), calcium, and phosphorus participate in LVH and affect myocardial contractility in end-stage renal disease. Several investigators have found that myocyte hypertrophy and increased interstitial collagen matrix in renal failure are related to PTH concentrations[5,24] and PTH could influence LVH in chronic dialysis patient patients when it is remarkably elevated.[25] Furthermore, correction of secondary hyperparathyroidism (SHPT) with intravenous calcitriol results in regression of LVH in HD patients, without biochemical or hemodynamic changes.[26] Hypertension in hemodialysis patients is associated with concentric hypertrophy of left ventricle.[13,27] Likewise, LVM indices are significantly higher than their normotensive counterparts but these indices are similar to those non-uremic hypertensive patients, demonstrating that inadequate blood pressure control is an important factor for development of LVH.[14]

Thus secondary hyperparathyroidism does play a role in the cardiovascular risk in end stage renal disease (ESRD).[1,2] However, previous studies evaluating the role of PTH in LVH in ESRD were conflicting.[2,5–7] The genesis of hypertension in hemodialysis patients is multi-factorial. Raisedintracellular calcium as a consequence of secondary hyperparathyroidism might add to the risk of hypertension in hemodialysis patients.[28,29] Moreover, hypertension is associated with an increased risk for left ventricular hypertrophy, coronary artery disease, congestive heart failure, cerebrovascular complications, and mortality. Association of secondary hyperparathyroidism and hypertension is commonly observed in hemodialysis patients. Therefore, this association might contribute in escalating LVH.

Our study showed that LVM and LVMI were significantly higher in patients with markedly high iPTH. Furthermore, in concurrence with the others, our data showed that LVM index values correlated positively with iPTH and mean arterial blood pressure.[30]

Our findings clearly demonstrated that LVM and LVMI were significantly higher inpatients with concomitant high BP and high iPTH. Furthermore LVMI was significantly high in patients with high iPTH alone. These findings are ambiguously demonstrated in the literature. Eminently, LVMI was high in Group 2 (mean BP less than 107 mmHg with iPTH more than 32 pmol/l) and higher in Group 4 (mean BP more than 107 mmHg with iPTH more than 32 pmol/l). These findings could mean that secondary hyperparathyroidism plays a partial role in inducing LVH even with controlled blood pressure. Concomitant hypertension and hyperparathyroidism add up effect in developing left ventricular hypertrophyin hemodialysis patients. Treatment of secondary hyperparathyroidism with intravenouscalcitriol significantly reduced myocardial hypertrophy in HD patients with SHPT andshortened QT dispersion, without hemodynamic or biochemical changes.[31] The newly introduced non-aluminum, non-calcium-based phosphorus binders, such as sevelamer hydrochloride[32] and lanthanum carbonate,[33] as well as calcimimetic agents[34] represent a breakthrough in the management of hyperphosphatemia and secondary hyperparathyroidism. A limiting factor in this study is the fact that the association between MAP/iPTH and LVH was not controlled.

## Conclusion

Concomitant secondary hyperparathyroidism and high blood pressure are associated with LVH in patients undergoing hemodialysis. Secondary hyperparathyroidism on its own might also contribute in escalating LVH. More awareness for adequate control of blood pressure and hyperparathyroidism might reduce the risk of developing LVH. Further studies of larger scale are required to confirm this finding.
